# Low Luteal Serum Progesterone Levels Are Associated With Lower Ongoing Pregnancy and Live Birth Rates in ART: Systematic Review and Meta-Analyses

**DOI:** 10.3389/fendo.2022.892753

**Published:** 2022-06-10

**Authors:** Noemie Ranisavljevic, Stephanie Huberlant, Marie Montagut, Pierre-Marie Alonzo, Bernadette Darné, Solène Languille, Tal Anahory, Isabelle Cédrin-Durnerin

**Affiliations:** ^1^Department of Reproductive Medicine, Centre Hospitalier Universitaire (CHU) and University of Montpellier, Montpellier, France; ^2^Department of Reproductive Medicine, Centre Hospitalier Universitaire (CHU) Carémeau, Nîmes, France; ^3^Center for Human Reproduction-Institut Francophone de Recherche Et d’études Appliquées à la Reproduction Et Sexologie (IFREARES), Clinique Saint Jean du Languedoc, Toulouse, France; ^4^Gedeon Richter France, Paris, France; ^5^Monitoring Force, Maisons-Laffitte, France; ^6^Department of Reproductive Medicine, Hôpital Jean-Verdier, Bondy, France

**Keywords:** luteal progesterone concentration, corpus luteum, hormonal replacement therapy, ongoing pregnancy, live birth, IVF

## Abstract

Progesterone plays a key role in implantation. Several studies reported that lower luteal progesterone levels might be related to decreased chances of pregnancy. This systematic review was conducted using appropriate key words, on MEDLINE, EMBASE, and the Cochrane Library, from 1990 up to March 2021 to assess if luteal serum progesterone levels are associated with ongoing pregnancy (OP) and live birth (LB) rates (primary outcomes) and miscarriage rate (secondary outcome), according to the number of corpora lutea (CLs). Overall 2,632 non-duplicate records were identified, of which 32 relevant studies were available for quantitative analysis. In artificial cycles with no CL, OP and LB rates were significantly decreased when the luteal progesterone level falls below a certain threshold (risk ratio [RR] 0.72; 95% confidence interval [CI] 0.62–0.84 and 0.73; 95% CI 0.59–0.90, respectively), while the miscarriage rate was increased (RR 1.48; 95% CI 1.17–1.86). In stimulated cycles with several CLs, the mean luteal progesterone level in the no OP and no LB groups was significantly lower than in the OP and LB groups [difference in means 68.8 (95% CI 45.6–92.0) and 272.4 (95% CI 10.8–533.9), ng/ml, respectively]. Monitoring luteal serum progesterone levels could help in individualizing progesterone administration to enhance OP and LB rates, especially in cycles without corpus luteum.

**Systematic Review Registration:**

https://www.crd.york.ac.uk/prospero/display_record.php?RecordID=139019, identifier 139019.

## Introduction

Progesterone plays a key role in implantation through several mechanisms such as endometrial differentiation (1), myometrial quiescence (2) or immune modulation (3). It has an essential function for the onset of pregnancy and is thus widely used in luteal support of assisted reproductive technique (ART) cycles.

Following the ovulation in a natural cycle, the predominantly oestrogen-secreting follicle is converted into a predominantly progesterone-secreting corpus luteum (CL). Progesterone secretion is sustained by the pituitary luteinizing hormone (LH) (4) through the luteal phase and is pulsatile with important variations through the day (5).

**Figure 1:**
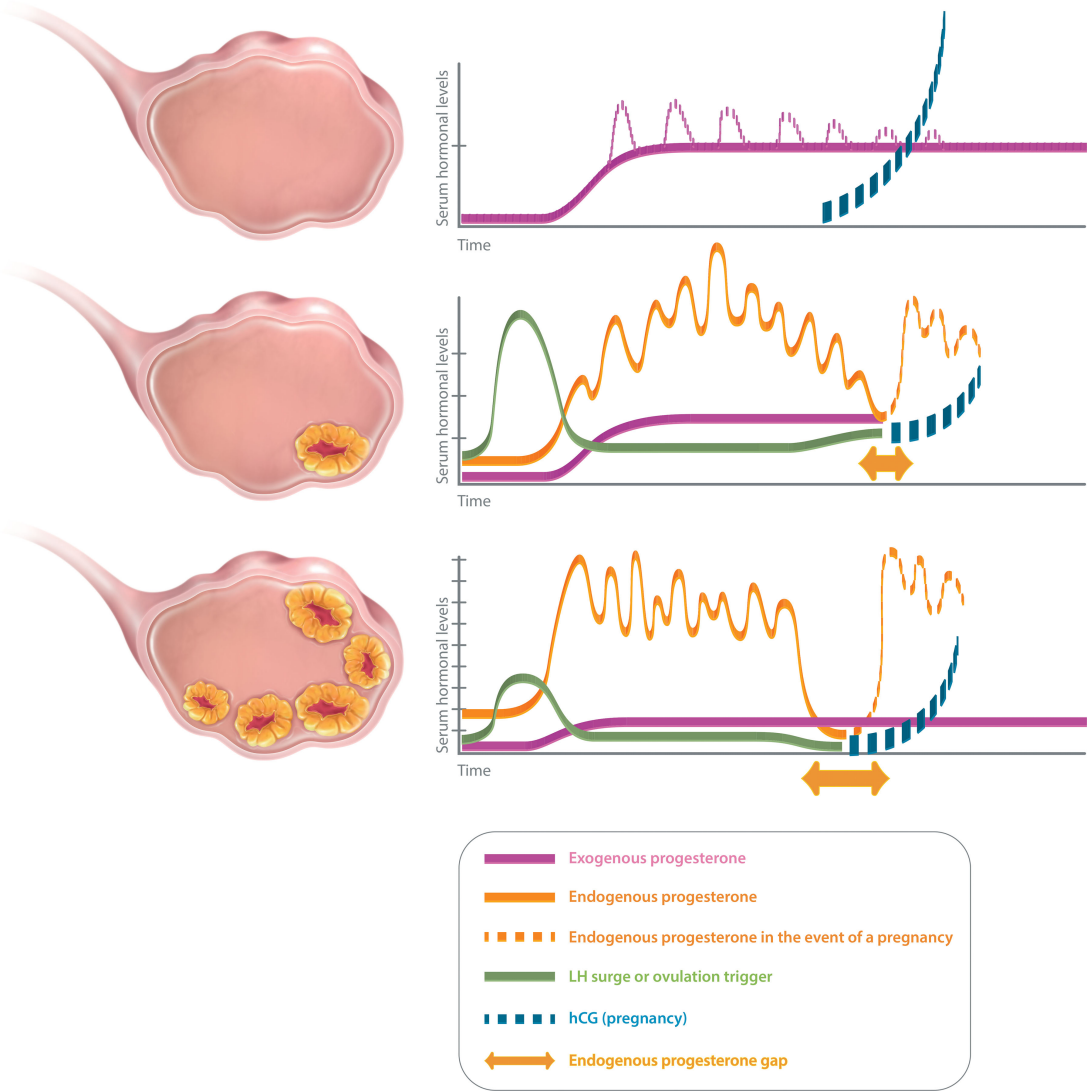
Luteal phase scenarios in ART. No corpus luteum (upper panel): artificial cycle (HRT). Progesterone only emanates from the luteal support (exogenous progesterone). Serum progesterone level quickly reaches a plateau (estimated serum progesterone level with vaginal progesterone: 10–15 mg/ml), except for injected progesterone for which peaks are observed initially (dotted line). Serum progesterone level is not modified in the event of a pregnancy as hCG does not interfere with the exogenous progesterone. One or few corpora lutea (middle panel): after ovulation trigger, endogenous progesterone secretion is pulsatile and varies during the day (estimated serum progesterone level: 25–35 mg/ml). There might be a small endogenous progesterone gap (mainly in cycles with few CL, in between a possible luteal insufficiency and the taking over of the hCG from the pregnancy). Luteal support is indicated to cover that gap. Several corpora lutea (lower panel): after ovulation trigger, endogenous progesterone secretion is pulsatile and varies during the day (estimated serum progesterone level: about 40–80 mg/ml, but might vary according to the number of CL). There will be a large endogenous progesterone gap (in between the iatrogenic luteal insufficiency and the taking over. Luteal support is indicated to cover that gap.

Different “luteal phase scenarios” may be encountered in ART practice, according to the number of corpus lutea (**Figure 1**). In an artificial cycle (no CL), the only source of progesterone is exogenous. Hence, the progesterone concentration measured during the luteal phase of an artificial cycle is the reflection of progesterone administration. In a stimulated cycle (one to multiple CLs), the progesterone level during the luteal phase results not only from the progesterone administration but also and mainly from the CL secretion, as long as the stimulatory effects of human chorionic gonadotropin (hCG) remain. However, due to the “luteal gap” between the stimulatory effects of exogenous hCG—used for triggering ovulation—and endogenous hCG originating from the conceptus (6), the luteal phase of a stimulated cycle with several CLs is deficient (7), possibly due to the multi-follicular development and the supra-physiological steroid levels which directly inhibit the pituitary LH release *via* a negative feedback (8) and cause premature luteolysis. A progesterone supplementation is thus necessary to maintain a sufficient progesterone level, until the implanted blastocyst takes over with its own hCG secretion to stimulate the CL progesterone secretion during early pregnancy.

Therefore, the usefulness of luteal support in ART cycles is commonly admitted (9). However, it is only recently that some authors (10, 11) suggested that monitoring the efficiency of this luteal support, through measurements of serum progesterone level during the luteal phase, might be of interest, as they reported a correlation with the pregnancy, miscarriage and live birth rates.

This systematic review and meta-analysis aims at evaluating the association between the luteal serum progesterone levels and the ongoing pregnancy (OP) and live birth (LB) rates (primary outcomes) as well as the miscarriage rate (secondary outcome), at different time points of the luteal phase, with various luteal support types and according to the number of corpora lutea (CL): none [artificial cycle for frozen-thawed embryo transfer (FET)], one or few (ovulation induction, intrauterine insemination, natural cycle for FET) or several (fresh embryo transfer).

## Methods

### Literature Search Strategy and Eligibility Criteria

This study was conducted using the methods of the Cochrane Collaboration (12) registered *a priori* in the International Prospective Register of Systematic Reviews (PROSPERO: CRD42019139019). The PRISMA (Preferred Reporting Items for Systematic Reviews and Meta-analyses) checklist was used while writing this review.

Randomised controlled trials and cohort and case–control studies were included if they (i) involve infertile women undergoing ovulation induction, intrauterine insemination, *in vitro* fertilization/intracytoplasmic sperm injection (IVF/ICSI) or frozen embryo transfer and (ii) reported serum progesterone levels (ng/mL or nmol/L; 1 ng/ml = 0.314465 nmol/l) determined during the luteal phase and clinical outcomes (pregnancy or live birth rates). The primary outcomes of interest were the OP rate per transfer (OPR, as defined by the number of viable intrauterine pregnancies of 12 weeks of gestation or more divided by the number of transfers) (13) and the LB rate per transfer (LBR, as defined by the number of deliveries of a living infant after 22 weeks of gestation divided by the number of transfers) (14). The secondary outcome was the miscarriage rate per transfer (MR, as defined by the number of intrauterine pregnancy losses before 12 weeks of gestation in size on ultrasound divided by the number of transfers) (11).

Studies were excluded from the review if they (i) reported follicular serum progesterone concentration measurement only, or serum progesterone concentration measurement during pregnancy only, (ii) performed the measure of serum progesterone at the time of pregnancy test only except for artificial cycles (no corpus luteum interfering with measurement) and (iii) involved luteal phase support modification without progesterone measure afterward.

The electronic databases PubMed, Embase and the Cochrane Library were searched for publications from 1 January 1990 to 1 March 2021. The search strategy was limited to articles published in English or French involving human subjects. The research was developed in association with the referral Inter-University Library of Medicine of Paris Descartes, Paris 5, France. The searches were performed using a combination of Medical Subject Headings (MeSH) and free text terms for the following search terms (and their variants): ‘ART’, ‘IVF’, ‘ICSI’, ‘IIU’, ‘ovulation induction/stimulation’, ‘embryo transfer’, ‘progesterone’, ‘follicular phase’, ‘live birth’ and ‘pregnancy’ (**Supplementary Data 1**).

### Study Selection and Data Extraction

Two independent reviewers, blind to authors, institutions, journal titles and study results, performed an initial screening of the title and abstract of all articles.

Based on the pre-established inclusion criteria, the full texts of all remaining articles were assessed for inclusion by two independent reviewers. Any disagreement or uncertainty was resolved by discussion among reviewers to reach a consensus. A third independent reviewer solved any persisting disagreements. The methodological quality of the selected studies was assessed using the Cochrane Handbook methods, adjusted to study specific requirements (15). Outcome selection and measurement were assessed for three distinct outcomes (OPR, MR, LBR). Risks of bias were assessed by two independent reviewers using ROBIN-1 tools (16). Each risk of bias criteria was judged as ‘low’, ‘high’ or ‘unclear’ risk.

Data were extracted from included articles by two independent reviewers out of six reviewers using a data extraction form developed for the present review (**Supplementary Data 2**). When certain information/data were missing, the original author was contacted. Failing an answer from the study authors, articles with outcomes expressed only as percentages were excluded.

### Data Synthesis and Meta-Analyses

The software Review Manager 5.3.5 (Copenhagen: The Nordic Cochrane Centre, The Cochrane Collaboration, 2014) was used to combine and analyse the aggregated data. Independent meta-zanalyses were performed according to the number of CLs: several (fresh embryo transfer), one or few [ovulation induction, intrauterine insemination, natural cycle for frozen-thawed embryo transfer (FET)] or none (artificial cycle for FET). Each outcome was analysed independently.

By using continuous or dichotomous data, the summary statistics were the difference in means (MD) or the risk ratio (RR) alternatively, both with 95% confidence intervals (CIs). Using a random-effect model, we applied the inverse variance (IV) method. Pooled effect sizes were deemed statistically significant at p < 0.05. In addition to the estimation of between-study variance (Tau2), the Q chi-square test was used to test the heterogeneity between the studies. The inconsistency across studies was quantified using the I^2^ statistic and interpreted following the Cochrane Collaboration guide (17).

Forest plots were displayed by predefined subgroups: according to the route of progesterone administration for meta-analysis on artificial cycles with no CL, according to the type of ART for meta-analysis on cycles with one or few CLs and according to the time of progesterone determination for meta-analysis on cycles with several CLs.

The quality of evidence for each outcome was judged using the Grading of Recommendations Assessment, Development and Evaluation working group methodology (18).

### Sensitivity Analyses

Several prespecified sensitivity analyses planned on statistical analysis plan were performed on overall population and including subgroups. The estimates of fixed-effect meta-analysis were compared to random-effect models to check the robustness of the conclusions. Other sensitivity analyses assessed the potential impact of study weight and of the year of publication, by visual inspection of the forest plot displayed in ascending order of study weight and of year of publication. To verify whether the conclusion would have been different if the eligibility was restricted to studies with a low risk of bias, other sensitivity analyses were performed after omitting studies with at least one high risk of bias.

## Results

### Study Selection and Characteristics

**Figure 2:**
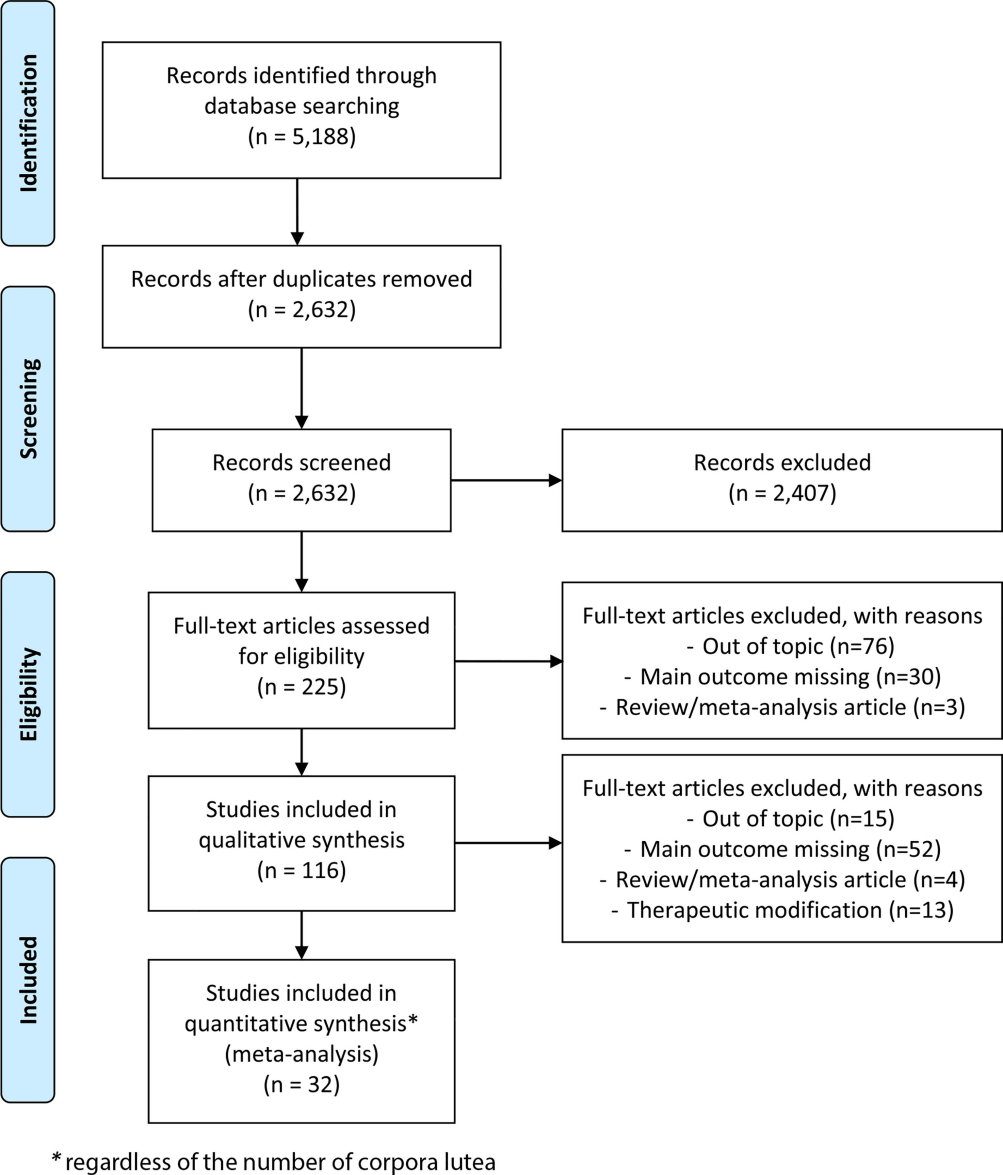
Study flowchart.

The search strategy identified a total of 5,188 articles, including duplicates and articles irrelevant to the primary research questions. After removing duplicates, 2,632 abstracts were reviewed, and 225 full-text articles were assessed for eligibility for quantitative analysis. Among them, 32 articles seemed potentially appropriate for inclusion in a meta-analysis, regardless of the number of CLs (**Figure 2**): this included 17 retrospective and 15 prospective studies, none of which were randomised trials.

The main characteristics of the included studies are reported in **Supplementary Table 1**. The included women were recruited during their first or second cycle (19–23), all of them (24–30) or not specified (11, 14, 31–46). Studies concerned either autologous cycles (13, 14, 19, 21, 22, 25, 27, 29–32, 36, 38–43, 45, 47), oocyte donation (20, 44) or not specified (11, 21, 23, 26, 28, 33–35, 37, 46). Studies included single-embryo transfers (11, 13, 22, 28, 33, 35, 40, 41), single- or double-embryo transfers (19, 20, 26, 27, 30, 31, 34, 37, 46), transfers of two to three embryos (24), transfers of three to four embryos (38, 39, 44) and not reported (14, 21, 23, 25, 29, 36, 43, 45, 47). Transferred embryos were cleaved embryos (21, 24, 27, 29, 34, 39), blastocyst embryos (11, 13, 14, 19, 20, 22, 23, 26, 31, 35, 40, 41, 46), both (28, 30, 33, 36, 37, 45) or not reported (38, 43, 44, 47). An ROC curve analysis was achieved in some studies, and luteal progesterone levels were predictive of pregnancy or live birth rates (19, 20, 25–27, 29, 32, 35, 36, 47), poorly predictive (24, 28) or not predictive (22, 45). Endometrial thickness was reported in some studies (14, 22, 25, 26, 28, 29, 36, 39). The number of retrieved oocytes for fresh cycles was reported in some studies (22, 25, 27, 30).

### No Corpus Luteum

In this luteal phase scenario, the meta-analysis included studies in which patients were categorised based on an arbitrary progesterone level on a certain day in the luteal phase (based for example on quartile, percentile and ROC curve; see discussion for threshold values). Results are thus presented as risk ratio (RR).

**Figure 3:**
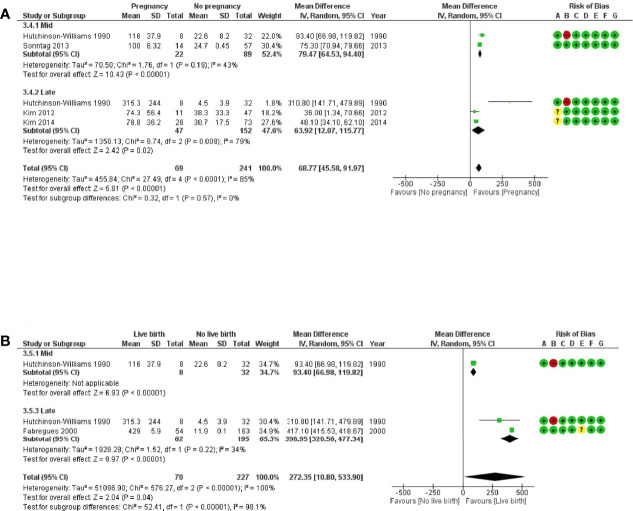
Forest plots of studies “no corpus luteum” according to the route of progesterone administration for **(A)** ongoing pregnancy and **(B)** live birth. Risk of bias legend: 1, confounding; **B**, selection of patients; **C**, classification of intervention; **D**, deviations from intervention; **E**, missing data; **F**, measurement of outcome; **G**, selection of reported results.

When comparing low to high luteal progesterone group, the RR (95% CI) for OP was 0.72 (0.62–0.84), confirming a significant negative association between low progesterone and OP (**Figure 3A**) (n = 3,501 cycles analysed from nine studies). Heterogeneity was moderate at 45%.

Subgroup analyses performed according to progesterone administration route (vaginal or injected or combined) showed that the heterogeneity was related to the only article reporting data on the injected route (35). The subgroup analysis on the vaginal route showed a null heterogeneity between the six studies and a significant negative association between low progesterone and OP [RR (95% CI) = 0.69 (0.62–0.77), n = 2,442 cycles analysed, I2 = 0%]. The subgroup analysis on the combined routes showed a null heterogeneity between the three studies and no significant association between low progesterone and OP [RR (95% CI) = 0.96 (0.76–1.21), n = 891 cycles analysed, I2 = 0%].

When comparing low to high progesterone group in artificial cycles with vaginal progesterone, the RR (95% CI) for LB was 0.73 (0.59–0.90), confirming a significant negative association between low progesterone and LB (**Figure 3B**) (n = 4,841 cycles analysed from eight studies). Heterogeneity was substantial at 78%, but all studies using vaginal progesterone only showed the same trend in favour of the high-progesterone group. The subgroup analysis on the combined routes showed no significant association between low progesterone and LB [RR (95% CI) = 0.98 (0.54–1.79), n = 520 cycles analysed, I2 = 85%].

When comparing low to high progesterone group, the RR (95% CI) for miscarriage was 1.48 (1.17–1.86), confirming a significant positive association between low progesterone and miscarriage (**Supplementary Figure 1**) (n = 2,918 cycles analysed from 13 studies). Heterogeneity was low at 33%.

### One or Few Corpora Lutea

In this luteal phase scenario, the meta-analysis included studies in which patients were categorised based on an arbitrary progesterone level on a certain day in the luteal phase. Results are thus presented as risk ratio (RR).

When comparing low to high progesterone group, the RR (95% CI) for LB was 0.60 (0.45–0.78), confirming a significant negative association between low progesterone and LB (**Supplementary Figure 2**) (n = 632 cycles analysed from three studies). Heterogeneity was low at 0%.

### Several Corpora Lutea

**Figure 4:**
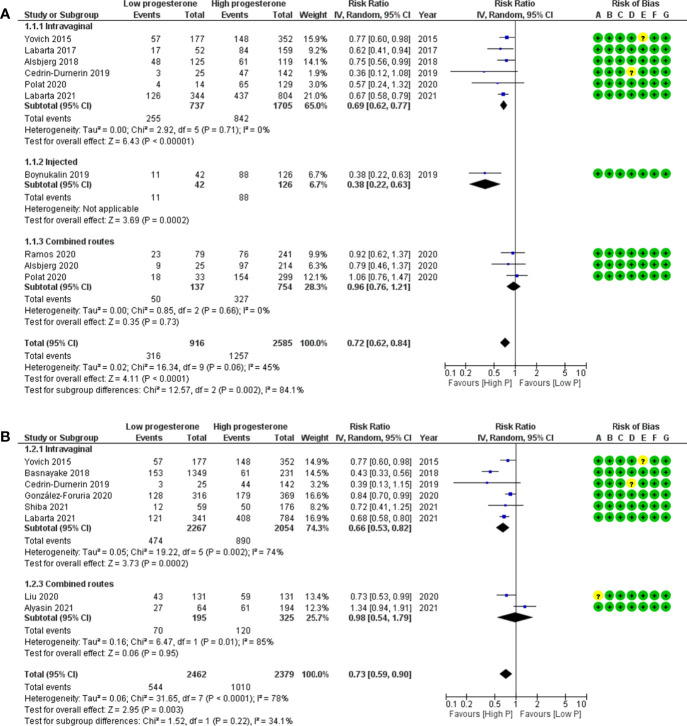
Forest plots of studies “several corpora lutea” according to time of progesterone determination for **(A)** ongoing pregnancy and **(B)** live birth. Risk of bias legend: 1, confounding; **B**, selection of patients; **C**, classification of intervention; **D**, deviations from intervention; **E**, missing data; **F**, measurement of outcome; **G**, selection of reported results.

In this luteal phase scenario, the meta-analysis included two types of studies: first, studies in which patients were compared depending on their cycle outcomes (pregnant or non-pregnant). Results are thus presented as the mean difference (MD) of progesterone levels (**Figure 4A**). Second are studies in which patients were categorised based on an arbitrary progesterone level on a certain day in the luteal phase (based on quartile, percentile, ROC curve, etc.). Results are thus presented as risk ratio (RR) (**Supplementary Figure 3**).

When comparing ongoing pregnancy to no pregnancy groups, the MD (95% CI) for the progesterone concentration was 68.8 (45.6–92.0) ng/ml, confirming a significant positive association between progesterone concentration and ongoing pregnancy (**Figure 4A**) (n = 310 cycles analysed from four studies). Heterogeneity was substantial at 85%. This could be observed either for midluteal progesterone concentration [MD (95% CI) = 79.5 (64.5–94.4) ng/ml, n = 111 cycles analysed from two studies, I2 = 43%] or for late-luteal progesterone concentration [MD (95% CI) = 63.9 (12.1–115.8) ng/ml, n = 199 cycles analysed from three studies, I2 = 79%].

When comparing live birth to no live birth groups, the MD (95% CI) for the progesterone concentration was 272.4 (10.8–533.9) ng/ml, confirming a significant positive association between progesterone concentration and live birth (**Figure 4B**) (n = 297 cycles analysed from two studies). Heterogeneity was substantial at 100%. This could be observed for the late-luteal progesterone concentration [MD (95% CI) = 399 (320.6–477.3) ng/ml, n = 257 cycles analysed from two studies, I2 = 34%].

When comparing low to high progesterone group, the RR (95% CI) for OP was 0.59 (0.32–1.07), showing no significant association between low progesterone and OP (**Supplementary Figure 3**) (n = 629 cycles analysed from three studies). Heterogeneity was moderate at 49%.

When comparing low to high progesterone group, the RR (95% CI) for LB was 0.87 (0.53–1.43), showing no significant association between low progesterone and LB (**Supplementary Figure 3**) (n = 1,172 cycles analysed from three studies). Heterogeneity was substantial at 74%.

### Secondary Analyses

Sensitivity analyses, even those after removing the study at high risk of bias (43), led to similar results and conclusions as main analyses. Patient selection was the only category of bias that was judged to be at high risk of bias in this study. Risks of bias are summarised in **Supplementary Figure 4**.

The quality of evidence of the GRADE approach is low and very low certainty of evidence on ‘no CL’ and ‘several CLs’ respectively (**Supplementary Table 2**).

## Discussion

This meta-analysis demonstrates that low luteal progesterone levels had a significant deleterious effect on ongoing pregnancy and live birth rates, in artificial cycles with no CL, in cycles with one or few CLs and possibly in stimulated cycles with several CLs.

Monitoring the luteal progesterone levels appears today as a fundamental step when managing patients undergoing ART procedures. However, it is important to keep in mind the main pitfalls in determining optimal serum progesterone levels. Serum progesterone levels vary according to the assay applied for its determination (48, 49). Hence, each clinic might need to determine its own threshold. Furthermore, progesterone levels might genuinely fluctuate over time. These variations are related to the administration route and the metabolism of the luteal support applied (exogenous progesterone), and to the pulsatility of the CL secretion (50) (endogenous progesterone) (**Figure 1**). It is thus important to distinguish in between the different luteal phase scenarios to interpret correctly the data.

### No Corpus Luteum

In artificial cycles (no CL), the progesterone only emanates from the luteal support of the hormonal replacement therapy (HRT) (exogenous progesterone). A large range of HRT protocols are available worldwide. The progesterone is most commonly administrated through the vaginal route, mainly in Europe (51) for its ease of use and because it increases the uterine progesterone concentrations by 10-fold (52) by avoiding the liver first-pass metabolism and through the uterine first-pass effect (53). The serum progesterone reaches its maximal concentration a few hours after the first vaginal administration and its plateau within the first 2 days (54–56). Hence, serum progesterone values in artificial cycles with vaginal progesterone can easily be interpreted as long as the measure is achieved after day 2 of progesterone. Serum progesterone concentrations reached after progesterone injections are higher (53), and fluctuations are frequent with serum peaks being observed at each injection for the first 4 days (57, 58), leading to a more complex interpretation of serum progesterone values until day 4 of progesterone. Hence, it is essential to consider the progesterone administration route and time of progesterone measure when interpreting progesterone values in the luteal phase of artificial cycles. Due to such differences in pharmacokinetics, we presented the data according to progesterone administration route. For all of these reasons, it is also delicate to define a minimal threshold for luteal progesterone for optimal pregnancy outcomes.

Women with low luteal progesterone levels had a significantly decreased chance of giving birth following FET with HRT (RR, 95% CI: 0.73, 0.59–0.90) when compared with women with high luteal progesterone levels.

In most studies using vaginal progesterone, the optimal luteal progesterone concentration seems to be above 8 to 15 ng/ml [7.8 ng/ml (28), 8.75 ng/ml (23), 8.8 ng/ml (26), 9.2 ng/ml (20), 10 ng/ml (41), 10.64 ng/ml (40), 11 ng/ml (31), 13.5 ng/ml (36) and 15.7 ng/ml (11, 33)]. More homogeneous thresholds between studies would probably allow a more accurate interpretation of the results. Some authors suggested that a luteal serum progesterone above 10 ng/ml may be a minimum required to ensure the best chance of implantation and ongoing pregnancy (59). However, too high progesterone levels might be associated with decreased implantation rate (11, 13). Some authors suggested that early high progesterone levels might desynchronise the uterine and embryo development, shifting the implantation window (60). One study was not included in the meta-analysis for missing data; from their analysis of 57 oocyte recipients using vaginal progesterone, the authors suggest that high progesterone concentrations are associated with failure to conceive or miscarriage (44). These conflicting conclusions might be explained by a very early measurement of luteal progesterone on the zygote intrafallopian transfer day in more than 50% of patients.

Results are more controversial in studies using injected progesterone for which the threshold might be around 20 ng/ml: on the one hand, some authors report a minimal threshold to be reached (35, 61); on the other hand, others define the optimal progesterone level below such threshold (60). However, in the latter, patients were excluded if their progesterone administration dosage was modified after progesterone level measurement on day 2 and progesterone level >10 ng/ml on the day of embryo transfer was required. This might bias the results as patients with low progesterone might have been thus excluded.

Women with low luteal progesterone levels had a significantly increased risk of miscarriage following FET with hormonal replacement therapy (HRT) (RR, 95% CI: 1.48, 1.17–1.86) when compared with pregnant women with high luteal progesterone levels. Thus, insufficient luteal progesterone concentrations are linked not only to a lower chance of pregnancy but also to a higher risk of miscarriage (37).

A substantial number of patients (30% to 50% depending on studies) seem to have an insufficient vaginal absorption of progesterone as reported by different authors (31, 36, 40, 62). Even if vaginal absorption could be influenced by external factors, such as sexual intercourses (63), and even if it suffers from a high inter-individual variability (11), it appears to be consistent from one cycle to another in about 80% of patients and a previous history of progesterone concentration value <10 ng/ml before embryo transfer is correlated with a low progesterone level on subsequent cycles (41, 62). It could thus be relevant to test vaginal absorption in patients planned for an HRT embryo transfer in a preceding cycle (64), in order to adjust the progesterone administration. Lower luteal progesterone concentrations are observed in obese patients, both with the vaginal (41) and the intramuscular route (65). Increasing vaginal progesterone doses might be an option for some patients; however, vaginal absorption might reach saturation (66, 67), which lessens the interest of such strategy for some patients (36). Studies are lacking to define the optimal progesterone dose increase to reach an optimal progesterone level in HRT using the intramuscular progesterone: an insufficient dose increase might not efficiently correct progesterone concentrations and pregnancy outcomes as suggested by some authors (10). However, some authors reported restored live birth rates after increasing the intramuscular progesterone and/or oestradiol administration for patients whose progesterone concentration 1 day prior to embryo transfer was below 15 ng/ml and/or oestradiol concentration was below 150 pg/ml (65).

Switching or combining the progesterone administration route (injected progesterone, oral dydrogesterone) might also be an option as suggested by some authors (68–71). Even a late correction of progesterone administration, the day before (68, 71) or the day of blastocyst transfer (70), seems to significantly improve pregnancy rates. This suggests that the mechanism behind implantation failure in the case of insufficient serum progesterone in HRT is probably not a simple endometrial receptivity defect. A recent study showed indeed no correlation between luteal serum progesterone and the endometrial receptivity assessed by the ERA test (72). The mechanism involved is not yet clearly defined and might be more than a faulty implantation window.

Moreover, our subgroup analysis on the combined routes showed no significant association between low progesterone and LB (RR, 95% CI: 0.98, 0.54–1.79), suggesting that multiple routes for progesterone administration could alleviate the lack of progesterone: patients with the lowest concentration would thereby not be deficient in progesterone, and their pregnancy rates would not be linked to their progesterone concentration anymore.

Unfortunately, the optimal progesterone threshold for combined administration routes has not been explored yet and is for now tricky to assess for oral dydrogesterone which does not cross-react with progesterone measurement. Dydrogesterone and its active metabolite 20α-dihydrodydrogesterone could be measured by liquid chromatography–tandem mass spectrometry. Some authors recently reported that their low serum levels on the embryo transfer day during an HRT cycle were associated with a lower ongoing pregnancy rate (73).

### One or Few Corpora Lutea

In natural cycles or in stimulated cycles with one or few CLs, interpreting the serum progesterone level is problematic as its secretion varies through a wide range during the day, with fluctuations up to eightfold within 90 min, more pronounced during the midluteal to late luteal phase (5). Women with low luteal progesterone levels had a significantly decreased chance of giving birth in a cycle with one or few CLs (RR, 95% CI: 0.60, 0.45–0.78) when compared with women with high luteal progesterone levels.

However, midluteal progesterone levels might depend on the follicle size (32) or might simply increase due to a higher number of CL as suggested by (74) who reported higher midluteal progesterone levels in intrauterine insemination cycles with a positive pregnancy test. Both studies included in our meta-analysis concluded that low midluteal progesterone is associated with a lower live birth rate (32, 42). Later progesterone level values might also be biased by the pregnancy itself: ovarian steroid secretion seems enhanced in conceptive cycles, due to a gonadotropic stimulus from the preimplantation embryo and due to the hCG secretion which stimulates the CL secretion (75). The midluteal and late luteal progesterone levels are thus difficult to analyse.

In contrast, secretion of progesterone in the early luteal phase was demonstrated to be independent of LH pulsatility (5). The profile of early luteal progesterone rise was reported to be associated with the length of the early luteal phase, which is largely variable among fertile women (76). In the natural FET cycle, measuring early luteal serum progesterone levels could be relevant to optimising synchrony between embryo and endometrial receptivity, as scheduling the embryo transfer on the early luteal serum progesterone concentration was reported to be more effective than scheduling it as usual on LH surge detection (77). The combination of the serum LH peak measurement and the early luteal serum progesterone rise could be an interesting option for clinicians wishing to optimise the timing of frozen-thawed embryo transfer in the natural cycle.

The only study included in our meta-analysis measuring early luteal serum progesterone levels reported its association with live birth rate (measured the day prior to blastocyst transfer in the true natural cycle without any luteal support) (14). However, when a GnRH agonist is used for both ovulation trigger and luteal support in a natural cycle, the early luteal progesterone levels do not seem to be related to pregnancy (78). One could hypothesise that a low early luteal progesterone could reveal a luteal phase deficiency, but the definition of such entity and its link with infertility are not clearly established (79). Another explanation could be that the synchrony between the endometrial window of implantation and embryo replacement is disrupted in patients with low early luteal progesterone.

### Several Corpora Lutea

In stimulated cycles with several CLs, live birth after a fresh embryo transfer in IVF (MD, 95% CI: 272.4, 10.8–533.9 ng/ml) was positively associated with a high progesterone concentration, especially for late luteal measures. However, these results need to be interpreted with caution: the confidence interval is extremely broad, and the analysis is pooling progesterone levels from the midluteal and late luteal phases. High late luteal progesterone might simply be a sign of rescued CL, when the conceptus secretes hCG which stimulates again the endogenous secretion of progesterone by the CL. Furthermore, there was no significant association between low progesterone and LB (RR 95% CI: 0.87, 0.53–1.43) for both early and midluteal progesterone measurements.

Luteal progesterone measured in stimulated cycles originates from the CL (endogenous progesterone) and from the luteal support (exogenous progesterone). The profile of the CL secretion is different depending on the ovulation trigger type: an earlier and more profound luteal phase deficiency is observed in cycles with a GnRH agonist trigger, even if it might greatly differ among patients (80).

However, in both trigger types, the progesterone concentration highly varies during the day: a single measurement might thus be difficult to interpret, except for low values, which seem to remain more stable (81), allowing the targeting of patients with deep luteal deficiency. Petersen et al. reported no live birth for patients whose progesterone levels were below 12.8 ng/ml at the time of pregnancy test (22). On the other hand, some authors suggested that there might be an upper threshold with lower chances of pregnancy with very high progesterone levels in the early and late luteal phases (39, 81).

If some authors concluded that there was no correlation in between pregnancy results and progesterone level on the embryo transfer day (22), others reported lower ongoing pregnancy or live birth rates with lower early luteal progesterone levels, with both vaginal progesterone and oral dydrogesterone support (27, 30). Fanchin et al. suggested that the utero-relaxing effect of progesterone on the non-pregnant uterus during the early luteal phase might contribute to the onset of pregnancy (39). However, Kim et al. did not account late luteal progesterone level (at pregnancy test) for a predictive factor of ongoing pregnancy in their retrospective study with 284 women using intramuscular progesterone support (25). These conflicting conclusions call for caution when interpreting the results of our meta-analysis. Furthermore, there could be important confounding biases as more follicles and oocytes are obtained in patients with higher luteal progesterone levels (30) and ovarian response was clearly demonstrated to be associated with live birth rate regardless of women’s age (82).

Nevertheless, a recent retrospective study reported that midluteal progesterone was associated with pregnancy and live birth rates in IVF agonist cycles (83). Interestingly, additional luteal support by dydrogesterone for patients with very low measurements would alleviate the pregnancy and live birth rates. Identifying patients with low luteal progesterone levels during a stimulated cycle might guide an individualization of the luteal support so as to optimise the chance of pregnancy.

### Limitations of the Study

This review and meta-analysis has some limitations. Most of the included studies are retrospective; therefore, the estimates are probably biased. Another limit is the inclusion of a small number of studies for the ‘one or few’ and ‘several CLs’ scenarios. Moreover, statistical heterogeneity was high in the meta-analyses, suggesting that potential confounding factors might influence the results. However, it has to be noted that a null heterogeneity was shown between the six studies reporting OP data using the progesterone vaginal route. Although the chosen thresholds differed from one study to the other, all results similarly conclude that low luteal progesterone levels in artificial cycles are associated with low ongoing pregnancy or live birth rates, except in combined routes. It may be that the different timing of progesterone measurement has little impact on the final result since, according to the pharmacokinetic data, all these measurements were performed at the plateau of serum progesterone, which is thought to be particularly stable on HRT. A possible confounding factor might explain both reduced vaginal absorption and the chance of pregnancy, such as an altered microbiota (84). Yet, similar results are also observed with injected progesterone, reinforcing thus the conclusions of a negative association with serum low progesterone level.

Despite the use of rigorous methodology including strict definition of the outcomes, the quality of evidence is low or very low. Hence, the conclusion of our meta-analysis needs to be disclosed with caution.

### Future Research Perspectives

Due to an increasing number of publications warning of obstetrical complications related to the absence of the corpus luteum (85, 86), the artificial cycle should probably be reserved for certain patients where no other alternatives are available for endometrial preparation. However, the artificial cycle remains the ideal setting to study the impact of serum luteal progesterone on pregnancy initiation, as its measurement is not disturbed by the corpus luteum and its highly changing endogenous secretion. If the association between luteal serum progesterone and pregnancy or live birth rates seems clearly established in HRT, more research is still needed to clarify the underlying pathophysiological mechanisms. Future research will aim at defining properly the optimal progesterone threshold depending on the progesterone administration route, especially in case of multiple administration routes.

There is still much to be explored for the individualization of luteal phase support in cycles with several corpora lutea. Some authors suggested closely monitoring the serum progesterone (‘luteal coasting’) so as to individualise the luteal phase support (87), but more trials are needed to precisely determine how to monitor serum luteal progesterone and when and how to adjust luteal phase support, differentiating cycles with hCG trigger and with GnRH agonist trigger. Identifying patients with previous report of poor absorption of vaginal progesterone could for instance allow better targeting of patients requiring individualization of their luteal support.

More clinical trials are also necessary to determine the best strategy: should clinicians optimise the individualization of luteal progesterone administration according to serum progesterone measurements? Should luteal support always be considered by a dual route to improve progesterone absorption without luteal progesterone monitoring? Or should one be cautious about the risk of administering excessive doses of progesterone (11)?

## Conclusion

Monitoring the luteal progesterone during an ART cycle is about to become unavoidable in our daily practice, so as to optimise our patients’ pregnancy and live birth rates. If the analysis of serum luteal progesterone is rather simple and reliable in artificial cycles with no CL (frozen embryo transfer in HRT cycles), it tends to be much less straightforward in cycles with CL(s) (fresh embryo transfer). More studies are needed to define the optimal progesterone threshold in the different CL settings and to determine the best strategies to reach it.

## Data Availability Statement

The original contributions presented in the study are included in the article/**Supplementary Material**. Further inquiries can be directed to the corresponding author.

## Author Contributions

NR was responsible for the study design and supervision, screening (titles, abstracts and full texts), quality assessment and data extraction; drafted and revised the manuscript; and validated the final version of the manuscript. SH, MM and P-MA completed the screening on full text, assessed the quality, extracted the data and revised the manuscript. BD provided methodological support and statistical expertise. SL provided methodological support, developed the search strategy, performed the literature search, analysed the data and drafted the manuscript. TA was responsible for the study design and supervision, screening (titles, abstracts and full texts), quality assessment and data extraction and revised the manuscript. IC-D was responsible for the study design and supervision, full-text screening, quality assessment and data extraction and revised and validated the final version of the manuscript. All authors contributed to the article and approved the submitted version.

## Funding

This work was sponsored by an unrestricted grant from Gedeon Richter France. The authors declare that this funder was not involved in the study design, collection, analysis, interpretation of data, the writing of this article or the decision to submit it for publication.

## Conflict of Interest

P-MA was employed by the company Gedeon Richter. BD and SL were employed by the company Monitoring Force.

The remaining authors declare that the research was conducted in the absence of any commercial or financial relationships that could be construed as a potential conflict of interest.

## Publisher’s Note

All claims expressed in this article are solely those of the authors and do not necessarily represent those of their affiliated organizations, or those of the publisher, the editors and the reviewers. Any product that may be evaluated in this article, or claim that may be made by its manufacturer, is not guaranteed or endorsed by the publisher.

## Abbreviations

ART, assisted reproductive technology; CL, corpus luteum; FET,frozen-thawed embryo transfer; HRT, hormonal replacement therapy; LB, livebirth; OP, ongoing pregnancy.
