# Supplementary material for: Low Luteal Serum Progesterone Levels Are Associated With Lower Ongoing Pregnancy and Live Birth Rates in ART: Systematic Review and Meta-Analyses
Source: Front Endocrinol (Lausanne). 2022 Jun 10;13:892753. doi: 10.3389/fendo.2022.892753 (PMC9229589; doi:10.3389/fendo.2022.892753)
Supplement: Supplementary file 1 [file DataSheet_1.docx]

Supplementary data 1 - MEDLINE search strategy

The MEDLINE search strategy was as follows:

("sperm injections, intracytoplasmic"[MeSH Terms] OR ("sperm"[All Fields] AND "injections"[All Fields] AND "intracytoplasmic"[All Fields]) OR "intracytoplasmic sperm injections"[All Fields] OR ("intracytoplasmic"[All Fields] AND "sperm"[All Fields] AND "injections"[All Fields]) OR "intracytoplasmic sperm injection"[All Fields] OR "icsi"[All Fields] OR "ivf"[All Fields] OR "fertilization in vitro"[MeSH Terms] OR ("fertilization"[All Fields] AND "vitro"[All Fields]) OR "fertilization in vitro"[All Fields] OR ("vitro"[All Fields] AND "fertilization"[All Fields]) OR "in vitro fertilization"[All Fields] OR "reproductive techniques, assisted"[MeSH Terms] OR ("reproductive"[All Fields] AND "techniques"[All Fields] AND "assisted"[All Fields]) OR "assisted reproductive techniques"[All Fields] OR ("assisted"[All Fields] AND "reproductive"[All Fields] AND "technology"[All Fields]) OR "assisted reproductive technology"[All Fields] OR "ovulation induction"[MeSH Terms] OR ("ovulation"[All Fields] AND "induction"[All Fields]) OR "ovulation induction"[All Fields] OR ("ovarian"[All Fields] AND "stimulation"[All Fields]) OR "ovarian stimulation"[All Fields] OR (intrauterine[All Fields] AND ("insemination"[MeSH Terms] OR "insemination"[All Fields])) OR "embryo transfer"[MeSH Terms] OR ("embryo"[All Fields] AND "transfer"[All Fields]) OR "embryo transfer"[All Fields] OR ("embryo"[All Fields] AND "transfers"[All Fields]) OR "embryo transfers"[All Fields])

AND ("progesterone"[MeSH Terms] OR "progesterone"[All Fields] OR "Progesterone/administration and dosage"[MeSH Terms])

NOT ("follicular phase"[MeSH Terms] OR "follicular phase"[All Fields])

AND ("live birth"[MeSH Terms] OR "live birth"[All Fields] OR ("live"[All Fields] AND "birth"[All Fields]) OR "delivery, obstetric"[MeSH Terms] OR ("delivery"[All Fields] AND "obstetric"[All Fields]) OR "obstetric delivery"[All Fields] OR "delivery"[All Fields] OR "pregnancy"[MeSH Terms] OR "pregnancy rate"[MeSH Terms] OR "pregnancy"[All Fields])

AND "1990/01/01"[PDAT] : "2021/03/01"[PDAT]

AND (English[lang] OR French[lang])

NOT "animals"[MeSH Terms:noexp]
