# Supplementary material for: Low Luteal Serum Progesterone Levels Are Associated With Lower Ongoing Pregnancy and Live Birth Rates in ART: Systematic Review and Meta-Analyses
Source: Front Endocrinol (Lausanne). 2022 Jun 10;13:892753. doi: 10.3389/fendo.2022.892753 (PMC9229589; doi:10.3389/fendo.2022.892753)
Supplement: Supplementary file 2 [file DataSheet_2.docx]

Supplementary data 2 - Collected data

The following study details were collected to characterize the included studies: country, study design, inclusion and exclusion criteria, type of ART, cycle rank; treatment type, endometrial preparation, triggering, type of luteal support, type of embryo transfer, stage and number of transferred embryos, progesterone concentration measurement (day, technique, threshold definition, ROC curve, threshold values, unit);

For low and high progesterone groups or any other category of progesterone concentration: number of subjects, endometrial thickness, oestradiol at triggering, number of oocytes if fresh IVF/ICSI, number of embryo transferred, number of follicles if IUI/OI, ongoing pregnancy (n, N, %), miscarriage (n, N, %), live birth per transfer (n, N, %).

For articles displaying data of live birth and no live birth groups: progesterone concentration [mean+/-SD or median (IQR)]

For articles displaying data of pregnant and non-pregnant groups: progesterone concentration [mean+/-SD or median (IQR)]
