# Supplementary figures and images for: Low Luteal Serum Progesterone Levels Are Associated With Lower Ongoing Pregnancy and Live Birth Rates in ART: Systematic Review and Meta-Analyses
Source: Front Endocrinol (Lausanne). 2022 Jun 10;13:892753. doi: 10.3389/fendo.2022.892753 (PMC9229589; doi:10.3389/fendo.2022.892753)

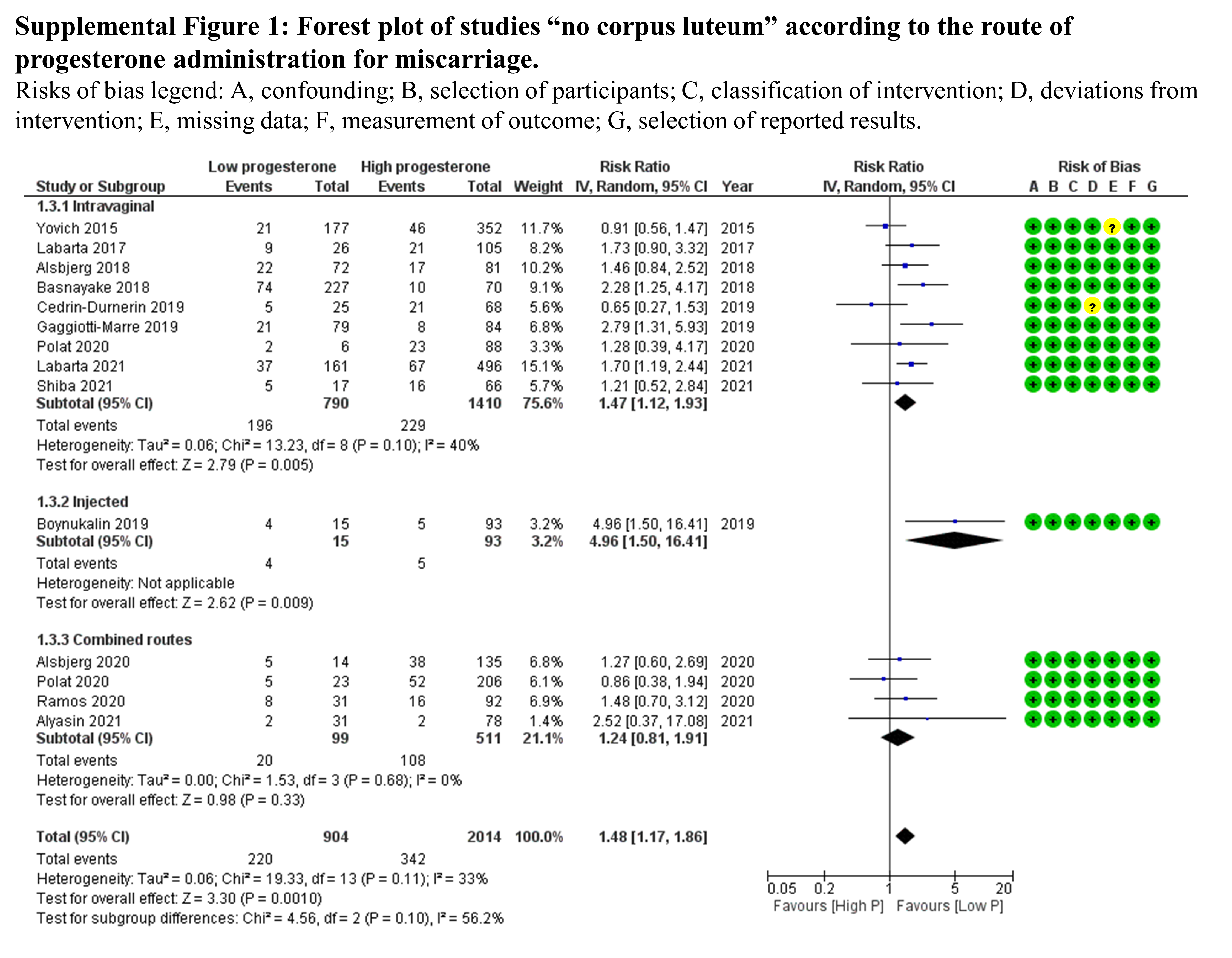

Supplement: Supplementary file 3 [file Image_1.tif]

Supplemental Figure 4: Risk of bias graph

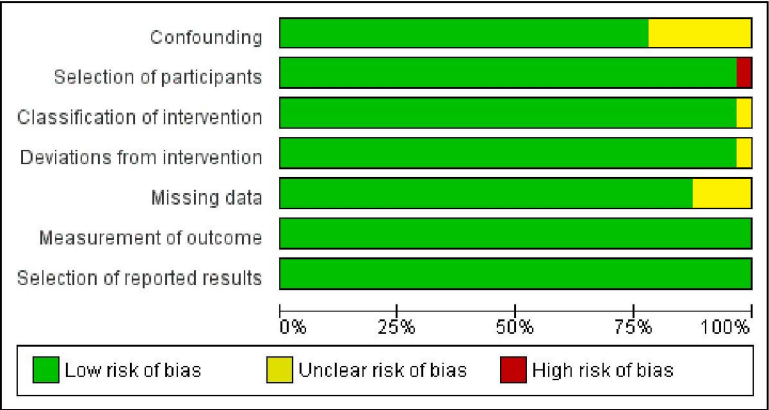

Supplement: Supplementary file 6 [file Image_4.pdf]
