# Supplementary material for: Low Luteal Serum Progesterone Levels Are Associated With Lower Ongoing Pregnancy and Live Birth Rates in ART: Systematic Review and Meta-Analyses
Source: Front Endocrinol (Lausanne). 2022 Jun 10;13:892753. doi: 10.3389/fendo.2022.892753 (PMC9229589; doi:10.3389/fendo.2022.892753)

Supplemental Figure 2: Forest plot of studies "one or few corpora lutea" according to the transfer type for live birth.

Risks of bias legend: A, confounding; B, selection of participants; C, classification of intervention; D, deviations from intervention; E, missing data; F, measurement of outcome; G, selection of reported results.

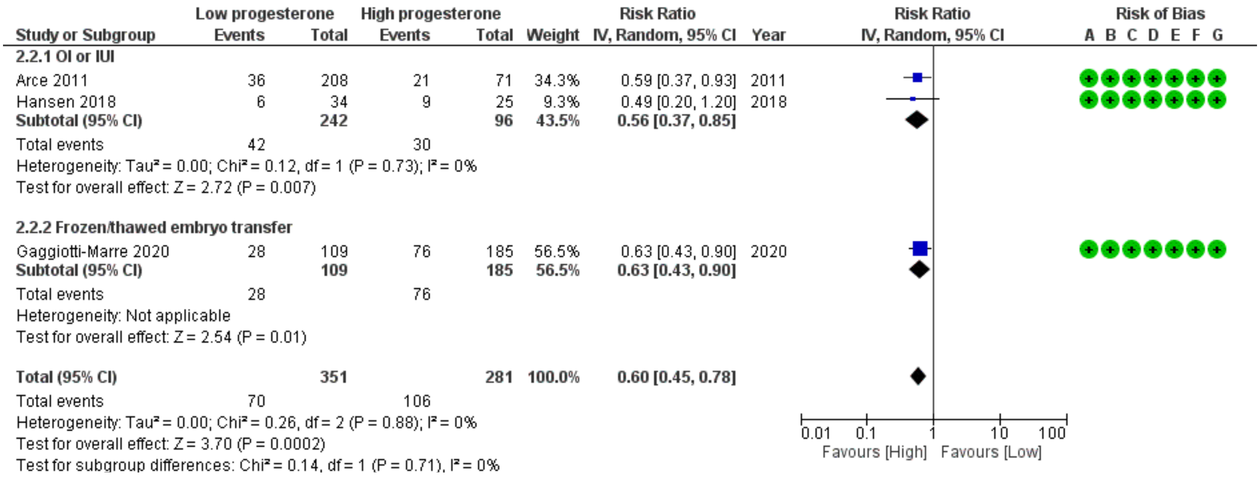

Supplement: Supplementary file 4 [file Image_2.pdf]
