# Supplementary material for: Low Luteal Serum Progesterone Levels Are Associated With Lower Ongoing Pregnancy and Live Birth Rates in ART: Systematic Review and Meta-Analyses
Source: Front Endocrinol (Lausanne). 2022 Jun 10;13:892753. doi: 10.3389/fendo.2022.892753 (PMC9229589; doi:10.3389/fendo.2022.892753)

Supplementary Figure 3: Forest plots of studies “several corpora lutea” according to time of progesterone determination for (A) ongoing pregnancy and (B) live birth.

Risks of bias legend: A, confounding; B, selection of participants; C, classification of intervention; D, deviations from intervention; E, missing data; F, measurement of outcome; G, selection of reported results.

(A)

	Low progesterone		High progesterone			Risk Ratio		Risk of Bias						
Study or Subgroup	Events	Total	Events	Total	Weight	IV, Random, 95% CI	Year	A	B	C	D	E	F	G
**3.1.1 Early**														
Fanchin 2000	6	34	9	25	27.0%	0.49 [0.20, 1.20]	2000	+	+	+	+	?	+	+
Netter 2019	3	50	40	192	20.0%	0.29 [0.09, 0.89]	2019	?	+	?	+	+	+	+
**Subtotal (95% CI)**		**84**		**217**	**47.0%**	**0.40 [0.20, 0.81]**								
Total events	9		49											
Heterogeneity: Tau² = 0.00; Chi² = 0.52, df = 1 (P = 0.47); I² = 0%														
Test for overall effect: Z = 2.56 (P = 0.01)														
**3.1.2 Mid**														
Benmachiche 2021	25	83	88	245	53.0%	0.84 [0.58, 1.21]	2021	?	+	+	+	+	+	+
**Subtotal (95% CI)**		**83**		**245**	**53.0%**	**0.84 [0.58, 1.21]**								
Total events	25		88											
Heterogeneity: Not applicable														
Test for overall effect: Z = 0.94 (P = 0.35)														
**Total (95% CI)**		**167**		**462**	**100.0%**	**0.59 [0.32, 1.07]**								
Total events	34		137											
Heterogeneity: Tau² = 0.14; Chi² = 3.89, df = 2 (P = 0.14); I² = 49%														
Test for overall effect: Z = 1.73 (P = 0.08)														
Test for subgroup differences: Chi² = 3.37, df = 1 (P = 0.07), I² = 70.3%														

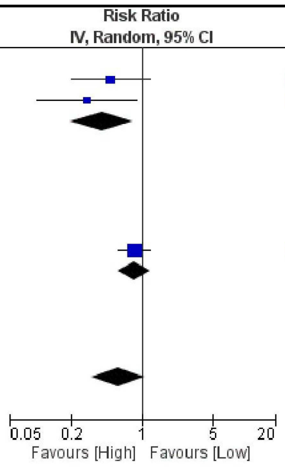

(B)

	Low progesterone		High progesterone			Risk Ratio		Risk of Bias						
Study or Subgroup	Events	Total	Events	Total	Weight	IV, Random, 95% CI	Year	A	B	C	D	E	F	G
**3.2.1 Early**														
Thomsen 2018	64	165	139	437	46.0%	1.22 [0.96, 1.54]	2018	+	+	+	+	+	+	+
Netter 2019	3	50	40	192	14.0%	0.29 [0.09, 0.89]	2019	?	+	?	+	+	+	+
**Subtotal (95% CI)**		**215**		**629**	**60.0%**	**0.66 [0.16, 2.68]**								
Total events	67		179											
Heterogeneity: Tau² = 0.87; Chi² = 5.99, df = 1 (P = 0.01); I² = 83%														
Test for overall effect: Z = 0.58 (P = 0.56)														
**3.2.2 Mid**														
Benmachiche 2021	25	83	85	245	40.0%	0.87 [0.60, 1.26]	2021	?	+	+	+	+	+	+
**Subtotal (95% CI)**		**83**		**245**	**40.0%**	**0.87 [0.60, 1.26]**								
Total events	25		85											
Heterogeneity: Not applicable														
Test for overall effect: Z = 0.75 (P = 0.45)														
**Total (95% CI)**		**298**		**874**	**100.0%**	**0.87 [0.53, 1.43]**								
Total events	92		264											
Heterogeneity: Tau² = 0.13; Chi² = 7.57, df = 2 (P = 0.02); I² = 74%														
Test for overall effect: Z = 0.55 (P = 0.58)														
Test for subgroup differences: Chi² = 0.14, df = 1 (P = 0.71), I² = 0%														

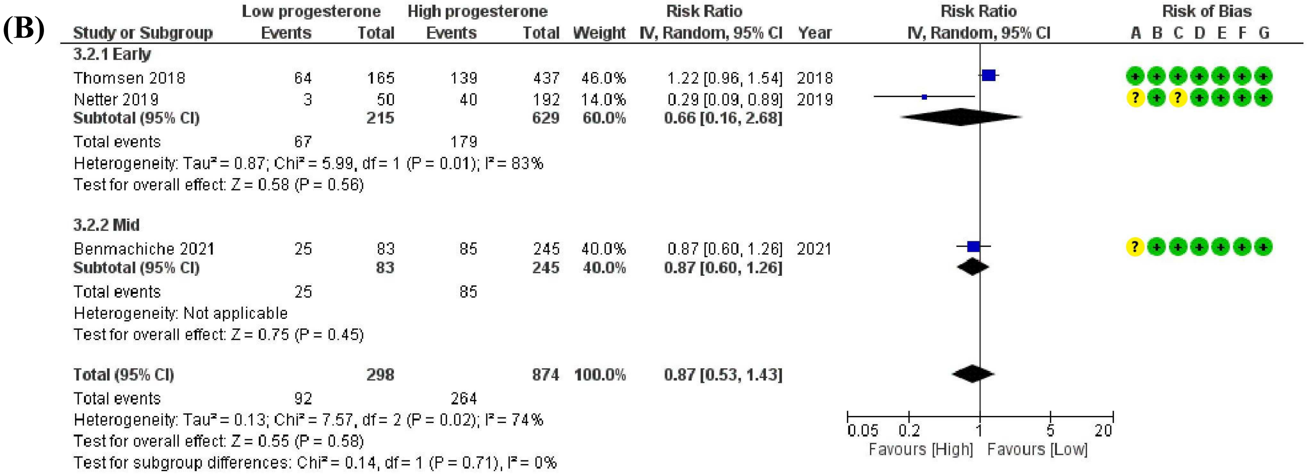

Supplement: Supplementary file 5 [file Image_3.pdf]
