# Supplementary material for: Low Luteal Serum Progesterone Levels Are Associated With Lower Ongoing Pregnancy and Live Birth Rates in ART: Systematic Review and Meta-Analyses
Source: Front Endocrinol (Lausanne). 2022 Jun 10;13:892753. doi: 10.3389/fendo.2022.892753 (PMC9229589; doi:10.3389/fendo.2022.892753)
Supplement: Supplementary file 7 [file Table_1.docx]

**Supplemental Table S1 Characteristics of studies included in the meta-analysis**

| CL status | Author, year, country | Design | Inclusion criteria | Exclusion criteria | Type of ART and Treatment | Luteal support | Day of Pg measurement (mid or late luteal phase) | Measurement technique | Threshold values (unit) |
| --- | --- | --- | --- | --- | --- | --- | --- | --- | --- |
| None | Johnson, 1995, UK | Retrospective | Oocyte recipients | Not reported | HRT | Vaginal Pg  300mg daily, pessaries | On the day of ET | ELISA kits (Boehringer® Mannheim Immunodiagnostics, Lewes, UK) | Not defined |
|  | **Yovich, 2015, Australia** | Retrospective | 22-49 years, SBET of defined grade (3BB or higher, according to the Gardner  grading system) | Not reported | HRT  Oestradiol valerate tablets (6mg daily). Then, 10mg oestradiol vaginal pessaries for 5 ± 1 days prior to Pg | Vaginal Pg  1200mg, pessaries.  With the evening pessary also containing oestradiol 2mg | On Day 8 or 9 of Pg administration | Immunoassay System ADVIA Centaur XP (Siemens, Erlangen, Germany; Cat# 10491445 – progesterone 250 test) | **15.7 ng/mL (50nmol/L)** |
|  | **Labarta, 2017, Spain** | Prospective | Oocyte recipients, <50 years, BMI <30 kg/m2, no systemic diseases, a triple layer endometrium >6.5 mm and 1–2 good quality Day 5 blastocysts | Recurrent miscarriages, implantation failure (RIF), severe male factor, uterine diseases or hydrosalpinx | HRT | Vaginal Pg, 800mg daily, pessaries | On the day of ET (Day 5) | Electrochemiluminescent immunoassay (Cobas® e 411 analyzer, Roche diagnostics GmbH, Germany) | **9.2 ng/mL** |
|  | **Alsbjerg, 2018, Denmark** | Retrospective | <45 years, BMI<35 kg/m2 | Oocyte donation, additional IM Pg, cleavage stage embryos | HRT | Vaginal Pg, 270mg daily, gel | 9 or 11 days after ET (at pregnancy test) | Electrochemiluminescent immunoassay (Cobas® Modular Analytics E170; Roche Diagnostics, Switzerland) | **11 ng/mL (35 nmol/L)** |
|  | **Basnayake, 2018, Australia** | Retrospective | Initial FET with HRT | No Pg concentration recorded at Day 16, treatment with ethinyl oestradiol, IM or rectal Pg | HRT | Vaginal Pg,  90 to 180mg daily, gel;  Vaginal Pg, 200 to 1200mg daily, pessaries. | On Day 16 post virtual OPU (pregnancy test) | Not reported | **15.7 ng/mL (50nmol/L)** |
|  | **Boynukalin, 2019, Turkey** | Prospective | HRT FET, with Day 5 hatching blastocysts which fully (100%) survived after warming procedure | Uterine diseases, hydrosalpinx, thin endometrium <7mm, embryos biopsied on day 6 | HRT | Intramuscular Pg 100mg daily | On the day of ET (Day 5) | Electrochemiluminescent immunoassay (Cobas® Elecsys Progesterone III, Roche diagnostics GmbH, Germany) | **13.6 ng/mL** |
|  | **Cedrin-Durnerin, 2019, France** | Retrospective | FET HRT | Oocyte donation program | HRT | Vaginal Pg 600mg daily, capsules | On the day of ET (Day 3 or 5) | Chemoluminescent immunoassays with an automated Elecsys immunoanalyser (ECLIA, Roche Diagnostics, Meylan, France) | **10 ng/mL** |
|  | **Gaggiotti-Marre, 2019, Spain** | Retrospective | Euploid FET | Uterine abnormalities, oocyte donation, mosaic embryos | HRT | Vaginal Pg 600mg daily, capsules | On the day before ET (Day 4) | Electrochemiluminescent immunoassay (Roche’s Cobas reagents in the Cobas e-411) | **10.64 ng/mL (median)** |
|  | **Alsbjerg, 2020, Denmark** | Prospective | Autologous embryos frozen on day 5 or 6, SET, BMI <34 kg/m2, and age 18-45 years. | Oocyte donation, supplemented with intramuscular Pg, uterine abnormalities, severe comorbidities | HRT | Vaginal Pg 180mg daily, gel  + rectal Pg 180mg daily, gel | 9 or 11 days after ET (at pregnancy test) | automated electrochemiluminescent immunoassays (Cobas® Modular analytics E170) (Roche Diagnostics, Rotkreuz ZG, Switzerland) | **8.8 ng/mL**  **(28 nmol/L)** |
|  | Commissaire, 2020, France | Retrospective | 18 to 43 years, BMI <32 kg/m2, French social security care | Not reported | HRT | Vaginal Pg 600 mg daily, pessaries | On the day of pregnancy test | Not reported | Not defined |
|  | **Gonzalez Foruria, 2020, Spain** | Retrospective | infertile women, <45 y-old, own oocytes, FET cycles | Not reported | HRT | Vaginal Pg, 600mg daily, pessaries (at 08:00, 16:00 and 00:00 h) | On the day prior to ET (blastocyste), and after 4 days of vaginal Pg administration, from 8am to 7pm | electrochemiluminescence immunoassay (Cobas® e-411 analyser, Roche Diagnostics, Germany). | **10 ng/mL** |
|  | **Liu, 2020, China** | Retrospective | age <48, undergoing two Day 2 or Day 3 embryo transfer, HRT | cryopreserved oocytes or donor oocytes, and with prior attempts at conception via IVF and FET | HRT | Intramuscular Pg 60 mg daily  + oral dihydrogesterone 30mg daily | 14 days after ET (at pregnancy test) | Not reported | **13.15 ng/mL (41.82 nmol/L)** |
|  | **Polat, 2020, Turkey** | Retrospective | 20 - 44 years, BMI 35 kg/m2 or lower, available day-5 or day-6 vitrified blastocyst(s) after warming. Only the first FET cycle. | PGT-A, PGT-M, PGT-SR | HRT | Vaginal Pg, 180mg, gel  OR  Vaginal Pg, 180mg, gel  + intramuscular Pg 50 mg daily from the first day of vaginal Pg administration, and from then onwards in the mornings before 9.00 am every third day (day 1, 4, 7, 10, 13) | On ET day (Day 5) | ImmunoDiagnostic Automated quantitative enzyme-linked fluorescent immunoassay (VIDAS Progesterone)(Bio-Merieux, Marcy I'Etoile, France). | **8.75 ng/mL** |
|  | **Ramos, 2020, France** | Retrospective | 18-42 years, 18<BMI<30kg/m2, morphologically normal uterus on hysterosonography and/or hysteroscopy | E2 priming superior to 28 days, stimulated cycles, recurrent pregnancy loss (ESHRE), congenital uterine abnormalities, endometrium thickness <7mm | HRT | Vaginal Pg, 800mg daily, capsules  + subcutaneous injections 25 mg daily | One or two days prior ET (blastocyst) | Chemiluminescence for the quantitative determination of the hormone serum level (Architect I2000, ABBOTT, Abbott Park, IL) | **14.53 ng/mL** |
|  | Al Jarrah, 2021, Iraq | Prospective | 20-40 years, BMI 19-35 kg/m2, normal uterine cavity and follopian tubes | RIF, pelvic pathology, thin endometrium <7mm basal Pg>1,5ng/ml, estrogen phase longer than 21 days | HRT | Vaginal Pg, 800mg daily, pessaries  +/- intramuscular Pg 50mg daily | On the day of ET (Day 3) | Compact automated immunoassay system (MINI VIDAS® BIOMÉRIEUX, France) by VIDUS PRG Kit | Not defined |
|  | Alyasin, 2021, Iran | Prospective | < 40 years, BMI< 30 kg/m2, 1st or 2nd FET using the FA strategy, with 1–2 top quality blastocysts | Oocyte or embryo donation cycles, recurrent miscarriages and implantation failure, severe male factor, uterine diseases or hydrosalpinx | HRT | Vaginal Pg suppository, 400mg than 800mg than 1200mg daily  + intramuscular Pg starting on the fourth day, 25mg then 50 mg daily | On the day of ET (blastocyst) | Enzyme-linked immunofluorescence assay (VITEK® ImmunoDiagnostic Assay System) | **19 ng/mL** |
|  | **Labarta, 2021, Spain** | Prospective | <=50 years old, with adequate endometrial pattern and thickness (6.5mm), LPS with only MVP (400mg twice daily for 5 days) before ET | Uterine or adnexal anomalies | HRT | Vaginal Pg,, 800 mg daily, capsules | On the day of ET (blastocyst) | Electrochemiluminescence immunoassay (CobasVR e411 analyzer, Roche diagnostics GmbH, Germany) | **8.8 ng/mL** |
|  | **Shiba, 2021, Japan** | Prospective | HRT FET | Contraindications listed on the medication package | HRT | Vaginal Pg, suppositories (4 different luteal phase support: 300mg Lutinus, 600mg Utrogestan, 800mg Luteum, 90mg Crinone) | On the day of ET (Day 2 or 3 or 5) | Electro-chemiluminescence immunoassay (Cobas e 411 analyzer; Roche Diagnostics GmbH, Germany) | **7.8ng/mL** |
| One or few | **Arce, 2011, Denmark** | Retrospective | Chronic anovulation; clomiphene-resistance; infertility for 1 year; 18–39 years; BMI 19–35 kg/m2; at least one patent tube; a normal pelvis at US scan; basal FSH 1 - 12 IU/l; normal prolactin and total testosterone not suggestive of androgen-secreting tumours; normal semen analysis | Mid-luteal Pg concentration < 7.9 ng/ml (25 nmol/l) | Low dose step up protocol, with either highly purified urinary menotropin, highly purified urinary FSH or rFSH.  Ovulation trigger with hCG 5,000 IU | No luteal support | 6 to 9 days after the hCG administration | Chemiluminescent immunoassay | **25 ng/mL**  (ranges from 7.9 to 40 and above) |
|  | **Hansen, 2018, USA** | Prospective | Unexplained infertility, 18-40 years, regular menses, normal uterine cavity with at least one patent tube | Not reported | (1) CC 100mg/D from D3 ± 2 for 5 days  (2) letrozole 5mg/D from D3 ± 2 for 5 days  (3) gonadotropin 150 IU/D from D3 ± 2, variable duration and dose  Ovulation trigger with hCG 10,000 IU | Not reported | 1 week (±1 day) after the IUI | Radioimmunoassay; Siemens Corporation | **15.8 ng/mL** for CC  **14.8 ng/mL** for letrozole  **9.1 ng/mL** for gonadotropin |
|  | **Gaggiotti-Marre, 2020, Spain** | Retrospective | Regular menstrual cycle, natural cycle FET with their own oocytes, serum Pg levels measured between 8 am and 11 am on the day before ET | Uterine abnormalities or mosaic ET, oocyte recipient  cycles and serum Pg extraction taken after 11 am | Natural cycle | None | On the day prior to ET (blastocyst) | Electrochemiluminescence immunoassay (CobasVR e-411 analyser; Roche Diagnostics, Germany) | **10 ng/mL** |
| Several | **Hutchinson-Williams, 1990, USA** | Retrospective | Normal ovulatory function, euprolactinemic | Not reported | hMG stimulation  Ovulation trigger with hCG 10,000 IU | A second dose of hCG (10,000 IU) 5 days later. | On day 13 and 16 (day 0 = ovulation trigger) | Radioimmunoassay using kits (Serono Laboratories, Inc., Randolph, MA) | Not defined |
|  | **Fabregues, 2000, Spain** | Prospective | 28-41 years, both ovaries, normal blood pressure and normal BMI, non-smoking, no medication, no intensive exercise | No fresh ET; no hCG for luteal support | Agonist protocol  Ovulation trigger with hCG 5,000 IU | hCG 2,500 IU on day 2 and day 5 | On day 13 or 14 after ET | Competitive chemiluminescent immunoassay (Immulite, DPC, Los Angeles, CA). | Not defined |
|  | **Fanchin, 2000, France** | Prospective | 23-38 years, normal uterus, at least 3 good quality embryos | Not reported | Agonist protocol with hMG  Ovulation trigger with hCG 10,000 IU | Vaginal Pg, 300mg daily | On the day of ET (day 2) | Radioimmunoassay using a I125 Progesterone Coatria kit (Bio-Merieux, Paris, France) | **100 ng/mL** (mean value) |
|  | **Kim, 2012, Korea** | Prospective | Infertile women with a fresh ET | Not reported | Agonist or antagonist protocol, rFSH  Ovulation trigger with hCG | Intramuscular Pg 50mg daily from OPU to week 6/7 | On day 14 after OPU | Electrochemiluminescence immunoassay (Elecsys Progesterone II; Hitachi High-technologies, Tokyo, Japan) | Not defined |
|  | **Sonntag, 2013, Germany** | Prospective | All infertile patients with a fresh ET | Frozen-thawed ET cycles; not applying rFSH under a long protocol | Agonist protocol with rFSH at 150 IU starting dose  Ovulation trigger with urinary hCG 10,000 IU or recombinant hCG 250mg | Vaginal Pg 600mg daily pessaries or 90mg daily gel | On day 7 after ET (day 2) | Chemiluminescence immunoassay (ECLIA, Roche, Mannheim, Germany) on Modular E170 automated analyser | Suggests **39.6 ng/mL (126 nmol/L)** |
|  | **Kim, 2014, Korea** | Prospective | Infertile women with a fresh ET | Not reported | Agonist or antagonist protocol, rFSH  Ovulation trigger with hCG | Intramuscular Pg 50mg daily from OPU to week 6/7 | On day 14 after OPU | Electrochemiluminescence immunoassay (Elecsys Progesterone II; Hitachi High-technologies, Tokyo, Japan) | Not defined |
|  | Kim, 2018, Korea | Retrospective | Infertile women with a fresh ET | Not reported | Agonist or antagonist protocol, rFSH  Ovulation trigger with hCG | Intramuscular Pg 50mg daily from OPU to week 6/7 | On day 14 after OPU | Electrochemiluminescence immunoassay (Elecsys Progesterone II; Hitachi High-technologies, Tokyo, Japan) | Suggests **29.8 ng/mL** |
|  | Petersen, 2018, Denmark | Prospective | 21–34 years, unexplained infertility or mild male factor infertility, BMI 18–25 kg/m2; FSH 1–12 IU/l, AFC 10 or over in total, and regular menstrual cycles of 24–35 days | Polycystic ovarian syndrome, endometriosis, poor response in a previous cycle | Antagonist protocol with hMG or rFSH at 150 IU starting dose  Ovulation trigger with recombinant hCG 250mg | Vaginal Pg 600mg daily, capsules | On ET day and on pregnancy test day | Electrochemiluminescence immunoassay (Roche Diagnostics ECLIA, Rotkreuz, Switzerland) | No live birth under **12.8 ng/mL** (on the day of pregnancy test) |
|  | Thomsen, 2018, Denmark | Prospective | < 41 years, BMI < 35 kg/m2 |  | Agonist or antagonist protocol with hMG or rFSH or rFSH+rLH  Ovulation trigger with hCG 5,000 to 10,000 or agonist | Vaginal micronized Pg 300mg daily from the day after OPU and until pregnancy test or up to week 7(hCG triggering)  hCG 1500 IU on OPU day and ± on OPU day+5 (agonist triggering) | On ET day (day 2/3 or 5) and on pregnancy test day (OPU+14) | Electro chemiluminescent immunoassays (Immulite® 2000XPi, Siemens Healthcare, Denmark and Architect® i2000SR, Abbott Diagnostics, USA) | Optimal range on day 5:  **47-78 ng/mL** **(150–250 nmol/l)** |
|  | **Netter, 2019, France** | Retrospective | Infertile women with a fresh ET | Not reported | Ovulation trigger with recombinant hCG 250 mg or dual trigger | Oral dihydrogesterone 30mg daily | On ET day (day 2-3) | Electrochemiluminescence immunoassay for Progesterone III  (Cobas 07092539 190) | **79 ng/mL (252 nmol/L)** |
|  | **Benmachiche, 2021, Algeria** | Retrospective | aged <40 years; baseline FSH < 12 IU/L; at least one embryo for fresh transfer | uterine fibroids, Mullerian malformations, ovarian , or adnexal abnormalities. | Antagonist protocol with rFSH, agonist trigger. | Bolus of HCG 1500 IU 1 hour after OPU + vaginal Pg 600 mg daily and estradiol orally 4 mg daily starting from the night of OPU  +/- additional single dose of GnRH-agonist on day OPU+6 | On ET day (day 2-3) | Vidas kit (BioMerieux, France) | **28 ng/mL** |
