# Supplementary material for: Low Luteal Serum Progesterone Levels Are Associated With Lower Ongoing Pregnancy and Live Birth Rates in ART: Systematic Review and Meta-Analyses
Source: Front Endocrinol (Lausanne). 2022 Jun 10;13:892753. doi: 10.3389/fendo.2022.892753 (PMC9229589; doi:10.3389/fendo.2022.892753)
Supplement: Supplementary file 8 [file Table_2.pdf]

Supplemental Table 2: GRADE tables for (A) « No corpus luteum », (B) « One or few corpora lutea » and (C) « Several corpora lutea »

**(A)**	**Risk of bias**	**Inconsistency**	**Indirectness**	**Imprecision**	**Publication bias**	**Certainty of evidence (GRADE)**
**Ongoing pregnancy**	Low	High	Moderate	High	Moderate	⊕⊕
**Live birth**	Low	Moderate	High	High	Moderate	⊕⊕
**Miscarriage**	Low	Low	High	High	Low	⊕⊕

**(B)**	**Risk of bias**	**Inconsistency**	**Indirectness**	**Imprecision**	**Publication bias**	**Certainty of evidence (GRADE)**
**Live birth**	Low	Moderate	Low	Low	Low	⊕⊕

**(C)**	**Risk of bias**	**Inconsistency**	**Indirectness**	**Imprecision**	**Publication bias**	**Certainty of evidence (GRADE)**
**Ongoing pregnancy**	Low	Moderate	Very low	Moderate	Very low	⊕
**Live birth**	Low	Moderate	Very low	Moderate	Very low	⊕
